# Investigation of Osteoporosis in Persons Living with Human Immunodeficiency Virus: The HOST Study

**DOI:** 10.1007/s00223-025-01368-8

**Published:** 2025-04-25

**Authors:** Simone Bruhn Rosendahl, Jakob Starup-Linde, Merete Storgaard, Bente Langdahl

**Affiliations:** 1 Department of Endocrinology and Internal Medicine, Aarhus University Hospital, Aarhus N, Denmark; 2 Department of Infectious Diseases, Aarhus University Hospital, Palle Juul-Jensens Boulevard 99, 8200 Aarhus N, Denmark

**Keywords:** Human immunodeficiency virus, Osteoporosis, Bone mineral density, High-resolution peripheral quantitative computed tomography

## Abstract

Bone health is an emerging concern in the aging human immunodeficiency virus (HIV)-infected population. We aimed to investigate bone mineral density (BMD) and bone microarchitecture in persons living with HIV (PLHIV). The study was a cross-sectional study. BMD and bone microarchitecture were investigated by Dual-Energy X-ray Absorptiometry (DXA) at the hip and lumbar spine and High-Resolution peripheral Quantitative Computed Tomography (HRpQCT) at the radius and tibia. Information about risk factors for fracture was obtained from a questionnaire. 183 PLHIV were included, 160 (131 males, 29 females) completed the visit. Age range was 30–78 years and the mean time since first HIV-positive serology was 16.5 years. 47% had low bone density and 6% had osteoporosis. The mean T-score by DXA was − 1.2 (standard deviation (SD) ± 1.0), − 0.7 (SD ± 0.9), and − 0.7 (SD ± 1.3) at the femoral neck, total hip and lumbar spine, respectively. We observed no significant difference in BMD by DXA between participants below or above age 50. BMI was significantly lower in PLHIV with lower BMD (p = 0.001, ANOVA). HRpQCT measurements showed significant differences in cortical area, volumetric BMD, and most microarchitecture parameters between PLHIV with BMD in the normal, low, and osteoporotic ranges measured at the tibia and radius. Our results indicate that bone microarchitecture and BMI vary considerably between PLHIV with normal, low, and osteoporotic BMD. These differences may partly explain the increased fracture risk seen in PLHIV.

*Trial numbers*: Ethics Committee of the Central Denmark Region (case no. 1-10-72-238-17), Danish Data Protection Agency (case no. 1-16-02-708-17).

**Supplementary Information:**

The online version contains supplementary material available at 10.1007/s00223-025-01368-8.

## Introduction

Human immunodeficiency virus (HIV) affects an estimated 38.0 million people worldwide [1]. Combined antiretroviral therapy (ART) has substantially reduced mortality and prolonged life expectancy for persons living with HIV (PLHIV), leading to an interest in age-related diseases as well as long-term effects of chronic HIV infection and ART treatment.

PLHIV have an increased risk of age-related diseases including osteoporosis suggested by a higher prevalence of reduced BMD and osteoporosis, respectively, compared to uninfected controls [2]. A meta-analysis report that PLHIV from a relatively young age of 40 years have a 35% increased risk of fragility fractures [3]. A register study of PLHIV found a three, nine and nine times greater risk of any, hip, and spine fracture, respectively [4]. Early intervention is therefore of great importance.

The etiological mechanism for bone loss in PLHIV is most likely multifactorial. Besides possible harmful effects of ART, HIV per se may deteriorate bone by events associated with T-cell repopulation in HIV disease reversal and dysregulation of the OPG/RANKL/RANK bone system [5, 6]. Also, traditional risk factors for low BMD including low BMI, high rates of tobacco and alcohol use and poor nutrition [7] may be more prevalent in PLHIV. Coinfection with hepatitis B and C virus is also an established risk factor for low BMD in PLHIV [8]. However, Fracture Risk Assessment Tool (FRAX) without BMD did not discriminate PLHIV with and without osteoporosis and thus common osteoporosis risk factors may not fully explain osteoporosis in PLHIV [9].

In addition to the above, deficits in BMD and microarchitectural bone changes as measured by High-Resolution peripheral Quantitative CT (HRpQCT) have also been visualized in young men infected with HIV early in life [10]. Knowledge of bone microarchitecture in PLHIV is, however, sparse. HRpQCT can discriminate between trabecular and cortical bone and estimate bone biomechanical properties including bone strength. HRpQCT volumetric BMD, microstructure, and failure load parameters can predict and discriminate incident fractures independently of each other and femoral neck (FN) areal BMD and FRAX [11, 12], indicating independent contribution to fracture risk. Previous studies using HRpQCT in PLHIV have investigated more narrowly defined groups, e.g., women [13], postmenopausal women [14], or young men [10]. Our volumetric density findings presented below are in line with findings in previous studies [10, 15–18] in HIV-infected men compared to healthy controls. Most of these studies primarily found alterations in the trabecular compartment. The findings of our study are especially in line with the findings in young HIV-infected men by Yin and colleagues [10]. Altered microarchitecture might result in and partly explain the increased fracture risk in PLHIV. A non-HIV study have found the HRpQCT parameters; cortical density, trabecular number, trabecular thickness at the radius and all of those along with cortical area at the tibia and failure load to be significantly associated with fracture risk in a large cohort study independent of aBMD and FRAX [12]. BMD is further reduced in PLHIV treated with ART compared to ART naïve [19]. ART initiation is associated with a 2–6% decrease in BMD at both the lumbar spine (LS) and total hip (TH) the first 1–2 years with stabilization hereafter [20, 21]. ART-related declines in BMD are more pronounced with tenofovir disoproxil fumarate (TDF)-based regimens compared to those not including TDF [20, 21].

The aim of this study was to investigate BMD and bone microarchitecture in people living with HIV.

## Methods

### Study Population and Design

The STROBE statement guideline for reports of cross-sectional studies was followed [22]. The study was approved by the Ethics Committee of the Central Denmark Region (case no. 1–10-72–238-17), Danish Data Protection Agency (case no. 1–16-02–708-17), and followed the Helsinki declaration. All participants provided written informed consent.

The study was designed as an observational longitudinal cohort study. Here, we report cross-sectional baseline data. Eligible participants were HIV-1-infected adults (≥ 18 years) with eGFR ≥ 40 mL/min and either age ≥ 40 years or two of the following: 1) hereditary predisposition to osteoporosis (direct line relative with osteoporosis), 2) previous fragility fracture, 3) current smoking, 4) excessive use of alcohol (> two standard units/day for men and > one standard unit/day for women), 5) systemic corticosteroid treatment, 6) early menopause (< 45 years), 7) BMI < 19 kg/m^2^, and 8) coinfection with hepatitis B virus (HBV) or hepatitis C virus (HCV). Participants were recruited between October 2017 and May 2018. Exclusion criteria included 1) current or former treatment with antiresorptives, bone anabolic drugs or lithium, 2) other bone diseases aside from osteoporosis, and 3) pregnancy.

### Questionnaire

Participants completed a questionnaire containing questions regarding ethnicity, hereditary predisposition for osteoporosis, tobacco and alcohol use, former or current HBV and/or HCV, menopausal status, glucocorticoid treatment, calcium and vitamin D supplementation, and previous fractures.

### Bone Mineral Density by Dual-Energy X-ray Absorptiometry, DXA

Participants underwent areal BMD (aBMD; g/cm^2^) assessment by DXA at the LS (L1-L4), FN and TH using two Hologic Discovery (Hologic Inc., Waltham MA, USA) bone densitometers. Ethnicity and sex of each participant were taken into consideration. The coefficient of variance for the scanners utilized have previously been reported to be 1.5% for the LS and 2.1% for the FN [23].

Osteoporosis was defined as by the WHO [24]: BMD sex-specific T-score by DXA at either the spine or hip of ≤ − 2.5 represent osteoporosis. We divided the low bone density group into two: − 1.0 < T-score < − 1.5 (mild low bone density) and − 1.5 ≤ T-score < − 2.5 (severe low bone density). PLHIV have a 1.5 time increased risk of any fracture compared to controls [4, 25]. As a decrease in T-score of 1.0 corresponds to a 1.5–2 time increased fracture risk in non-HIV-infected individuals, a T-score of − 1.5 (1.0 lower than the guideline cut-off for osteoporosis) was chosen as the cut-off between mild and severe low bone density [26].

### High-Resolution Peripheral Quantitative Computed Tomography, HRpQCT

At the distal radius and distal tibia, bone microarchitecture and volumetric BMD (vBMD; mg/cm^3^) were measured using a HRpQCT scanner (Extreme CT scanner, Scanco Medical AG, Brüttisellen, Switzerland). The participant’s arm and leg were immobilized in a carbon fiber shell during the scan. A reference line was defined at the distal endplate of the tibia and radius and 9.5 mm (tibia) and 22.5 mm (radius) proximal of this, the scanned regions started and were defined as the subsequent 9.02 mm. This gave in vivo three-dimensional quantification of trabecular and cortical structure of 110 CT slices at each site. Phantom scans for calibration were performed according to the manufacturer’s standard.

### Blood Sample and HIV Parameters

Latest CD4 cell count and mean time since first HIV-positive serology were obtained from the Danish HIV InfCare database, a database containing various HIV parameters for PLHIV in Denmark. Biochemical parameters obtained at clinical visits were obtained directly from electronic patient charts from the participants’ latest semiannual regular HIV outpatient control visits. These included creatinine, ionized calcium, and alanine transaminase (ALAT). In 5 participants, there were no previous biochemical parameters in the electronic patient chart. Bone markers C-terminal telopeptide of type 1 collagen (CTX) and procollagen type 1 N propeptide (P1NP) were measured in a fasting blood sample taken on the day of DXA and HRpQCT scans. CTX is a marker for bone resorption as the test is based on an antibody to the C-terminal end of the type 1 collagen fragments that enter the circulation when bone is resorbed by osteoclasts. P1NP is a bone formation marker as this reflects the N-terminal end of procollagen cleaved during bone formation.

### Statistical Analysis

All statistical analyses were made using STATA software, version 15.1 (StataCorp, College Station, TX, USA). Continuous data are presented as mean ± SD if normally distributed and median (interquartile range, IQR) if non-normally distributed. Means between two groups were compared using Student’s *t* test after checking normality. Differences in baseline characteristics and HRpQCT parameters between the four BMD groups were assessed by analysis of variance (ANOVA) test (if normally distributed) or Kruskal–Wallis test (if non-normally distributed) for continuous data and Fisher’s exact Chi-squared test for binary outcomes. Due to the multiplicity of testing (29 tests comparing DXA and HRpQCT measures between groups) we have Bonferroni corrected our P values to 0.0017 for significance. In secondary analyses, multiple regression was performed to adjust DXA measures and HRpQCT measures for age, sex, and BMI. T- and Z-scores were only adjusted by BMI as they inherently are adjusted by age and sex.

Because most guidelines for management of osteoporosis in PLHIV recommend screening in ≥ 50 years old males and postmenopausal women, we conducted sensitivity analyses where participants were grouped by age (< 50 or ≥ 50 years). P < 0.05 was considered statistically significant apart from the abovementioned Bonferroni correction.

## Results

**Fig. 1:**
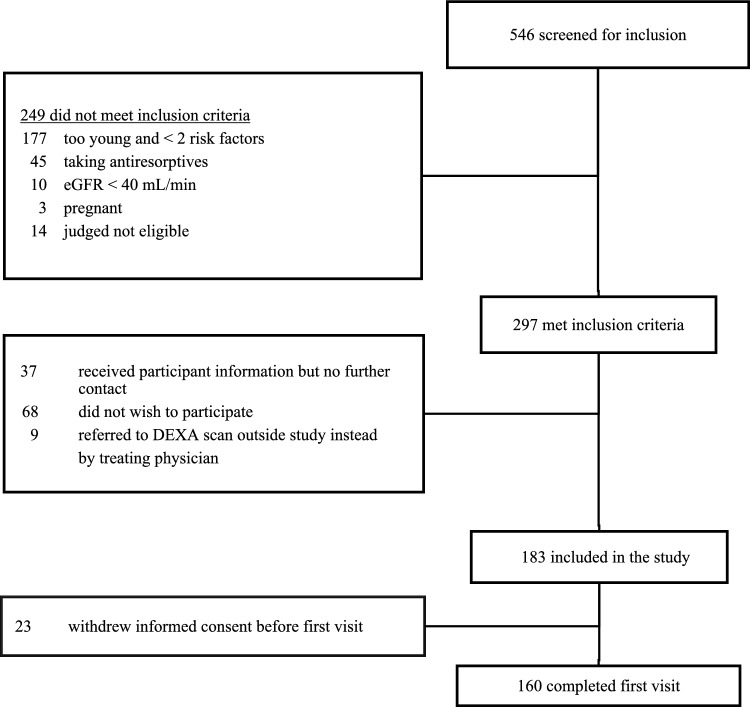
Flowchart of the inclusion and scan process

Flowchart of the screening and enrollment process is presented in Fig. 1. We accessed 546 PLHIV for eligibility. 297 met the inclusion criteria of which 183 (62%) accepted to participate. 160 completed the visit.

### Baseline Participant Characteristics

Table 1 shows participant characteristics. The study population was predominantly Caucasian (91%) and male (82%). Mean age was 56.2 (SD ± 8.4) years spanning from 30 to 78 years. The mean BMI was 25.7 (SD ± 4.3) kg/m^2^. 20 of the 29 females included were postmenopausal. Mean CD4 cell count was 690 (SD ± 332) and mean time since first HIV-positive serology was 16.5 (SD ± 9.4) years. 84% of participants had a history of treatment with TDF. Each BMD group and the study population in total showed normal ALAT and no hypo- or hypercalcemia.

**Table 1:** Participant characteristics. Presented as mean ± SD if normally distributed or median (IQR) if non-normally distributed. P values are derived from analysis of variance (ANOVA) if continuous variable and normally distributed, Kruskal–Wallis test if continuous variable and non-normally distributed and Fishers exact test if binary variable

	Normal	Mild low bone density	Severe low bone density	Osteoporosis	Total	p
N (%)	75 (47)	28 (18)	47 (29)	10 (6)	160 (100)	
Males, n (% in DXA group)	61 (81)	21 (75)	40 (85)	9 (90)	131 (82)	
Age (years)	56.4 ± 8.0	56.8 ± 7.5	55.8 ± 9.3	54.4 ± 10.3	56.2 ± 8.4	0.86
Ethnicity, n (% in DXA group)						
*Caucasian*	67 (89.33)	26 (92.86)	44 (93.62)	9 (90)	146 (91.25)	0.82
*Non-Caucasian*	8 (10.67)	2 (7.14)	3 (6.38)	1 (10)	14 (8.75)	
BMI (kg/m^2^)	26.7 ± 3.8	26.6 ± 5.1	24.6 ± 4.1	20.8 ± 2.6	25.7 ± 4.3	0.001
Mean time since first HIV-positive serology (years)	16.1 ± 9.7	16.5 ± 10.9	18.8 ± 7.9	18.6 ± 9.6	16.5 ± 9.4	0.88
TDF treatment ever, n (% in DXA group)*	60 (82)	23 (82)	41 (87)	8 (89)	132 (84)	
Smoking, n (%)						
Never	22 (29)	11 (39)	15 (32)	2 (20)	50 (31)	0.87
Former	34 (45)	12 (43)	18 (38)	5 (50)	69 (43)	
Current	19 (25)	5 (18)	14 (30)	3 (30)	41 (26)	
Hereditary predisposition, n (% in DXA group)						
Yes**	15 (20)	5 (17.86)	15 (31.91)	2 (20)	37 (23.13)	0.44
No***	60 (80)	23 (82.14)	32 (68.09)	8 (80)	123 (76.88)	
Creatinine (µmol/L)	83 ± 18	80 ± 17	83 ± 19	81 ± 19	82 ± 18	0.92
Ion calcium (mmol/L)	1.25 ± 0.04	1.24 ± 0.04	1.25 ± 0.05	1.23 ± 0.04	1.25 ± 0.05	0.18
ALAT (U/L)	29 ± 15	29 ± 22	28 ± 18	47 ± 76	30 ± 25	0.18
Latest CD4 cell count	662 ± 316	729 ± 328	695 ± 329	771 ± 483	690 ± 332	0.68
Bone markers						
*CTX (ng/mL)* (n = 119)****	0.354 (0.238–0.489) (n = 58)	0.417 ± 0.149 (n = 19)	0.455 ± 0.181 (n = 34)	0.584 ± 0.204 (n = 8)	0.429 ± 0.183 (n = 119)	0.05
*P1NP (ng/mL)*	51.19 (39.21–66.48)	64.50 ± 32.85	59.49 ± 23.91	71.87 ± 26.76	60.08 ± 27.48	0.24

^*^ Treatment data missing for three participants

^**^ First-line relative with vertebral fracture, hip fracture or osteoporosis

^***^ No first-line relative with vertebral fracture, hip fracture, or osteoporosis

^****^ Only reported for the 119 participants who were fasting

Unfortunately, 41 participants (26%) were not fasting when having their blood sample taken. As this can affect especially CTX values, we analyzed CTX and P1NP values in fasting, non-fasting, all participants, and the total BMD group. Testing showed significant difference in CTX levels (p = 0.004), but not P1NP levels (p = 0.71), between the fasting and non-fasting group (see supplemental Table 1). We therefore excluded non-fasting participants from the analysis of CTX. CTX (only those fasting) and P1NP (total group of participants) levels were 0.429 (SD ± 0.183) and 60.08 (SD ± 27.48) ng/mL, respectively.

### DXA

DXA results are presented in Table 2. 47% had normal BMD, 47% low bone density (18% mild low bone density, 29% severe low bone density), and 6% osteoporosis at the LS and/or TH. Mean T-scores were − 1.2 (SD ± 1.0), − 0.7 (SD ± 0.9), and − 0.7 (SD ± 1.3) for the FN, TH, and LS, respectively. No significant differences in DXA parameters between male and female participants or between pre- and postmenopausal female participants was observed (data for the latter not shown). For participants < 50 years, mean Z-scores for males were − 0.44, − 0.45, and − 0.53 for the FN, TH, and LS, respectively, and mean Z-scores for females were − 0.92, − 0.64, and − 0.50 for the FN, TH, and LS, respectively (Table 3 and 4). No significant difference in BMD by DXA was found in males or females between participants < 50 years and participants ≥ 50 years at any scan site (Table 3 and 4).

**Table 2:** Results of DXA scan. Presented as mean ± SD. P values are derived from Student’s *t* test of difference in BMD, Z-, and T-score between male and female participants. P adjusted with BMI adjustment

	Male (n = 130 for FN and TH, n = 131 for LS)	Female (n = 29)	Total (n = 159 for FN and TH, n = 160 for LS)	p	P adjusted
Femoral neck (FN)					
aBMD (g/cm^2^)	0.757 ± 0.133	0.751 ± 0.159	0.756 ± 0.138	0.84	0.49
Z-score	− 0.4 ± 1.0	− 0.3 ± 1.2	− 0.4 ± 1.0	0.54	0.28
T-score	− 1.3 ± 1.0	− 1.1 ± 1.1	− 1.2 ± 1.0	0.43	0.81
Total hip (TH)					
aBMD (g/cm^2^)	0.916 ± 0.131	0.887 ± 0.139	0.911 ± 0.132	0.29	0.07
Z-score	− 0.3 ± 0.9	− 0.1 ± 1.0	− 0.3 ± 0.9	0.13	0.87
T-score	− 0.8 ± 0.9	− 0.6 ± 0.9	− 0.7 ± 0.9	0.44	0.71
Lumbar spine (LS)					
aBMD (g/cm^2^)	1.013 ± 0.144	0.987 ± 0.125	1.008 ± 0.141	0.37	0.24
Z-score	− 0.2 ± 1.3	0.2 ± 1.2	− 0.1 ± 1.3	0.17	0.25
T-score	− 0.7 ± 1.3	− 0.8 ± 1.0	− 0.7 ± 1.3	0.84	0.65

**Table 3:** Scan results for male participants, stratified by age. Results presented as mean unless otherwise stated. CI, confidence interval. P values are derived from Student’s *t* test of difference in aBMD and vBMD between participants < 50 years or ≥ 50 years

	Age < 50 years (n = 27)	Age ≥ 50 years (n = 103 for FN and TH, n = 104 for LS)	Difference (95% CI)	P value
DXA				
*aBMD (g/cm*^*2*^*)*				
Femoral neck	0.787	0.749	0.038 (− 0.019; 0.094)	0.19
Total hip	0.926	0.913	0.012 (− 0.044; 0.069)	0.66
Lumbar spine	1.007	1.014	− 0.007 (− 0.069; 0.055)	0.81
*Z-score*				
Femoral neck	− 0.44	− 0.37	− 0.07	0.74
Total hip	− 0.45	− 0.32	− 0.13	0.50
Lumbar spine	− 0.53	− 0.08	− 0.45	0.12
HRpQCT				
*Total vBMD (mg/cm*^*3*^*)*				
Radius	326	299	26 (− 2;54)	0.07
Tibia	292	282	10 (− 14; 34)	0.39

**Table 4:** Scan results for female participants, stratified by age. Results presented as mean unless otherwise stated. CI, confidence interval. P values are derived from Student’s * t* test of difference in aBMD and vBMD between participants < 50 years or ≥ 50 years

	Age < 50 years (n = 10)	Age ≥ 50 years (n = 19)	Difference (95% CI)	P value
DXA				
*aBMD (g/cm*^*2*^*)*				
Femoral neck	0.718	0.769	− 0.050 (− 0.178; 0.078)	0.43
Total hip	0.853	0.905	− 0.052 (− 0.163; 0.059)	0.34
Lumbar spine	0.969	0.996	− 0.027 (− 0.128; 0.075)	0.59
*Z-score*				
Femoral neck	− 0.92	0.08	− 1.00	0.02
Total hip	− 0.64	0.22	− 0.08	0.01
Lumbar spine	− 0.50	0.06	− 1.07	0.02
HRpQCT				
*Total vBMD (mg/cm*^*3*^*)*				
Radius	312	290	2 (− 35;79)	0.45
Tibia	273	283	− 10 (− 59; 38)	0.66

Investigation of differences between BMD groups showed significant difference in BMI (p = 0.001), BMI was lower in PLHIV with lower BMD and a trend toward greater fasting CTX level in PLHIV with lower BMD (p = 0.05). P1NP was not associated with BMD levels.

### HRpQCT

HRpQCT results are presented in Tables 5 and 6. The mean total vBMD was 303 ± 66 mg/cm^3^ at the radius and 283 ± 56 mg/cm^3^ at the tibia as shown in Tables 5 and 6.

**Table 5:** Results of HRpQCT scan at radius site, presented as mean ± SD if normally distributed or median (IQR) if non-normally distributed. P values are derived from analysis of variance (ANOVA) if normally distributed and Kruskal–Wallis test if non-normally distributed. Padj adjusted by age, gender, and BMI

	Radius						
	Normal (n = 74)	Mild low bone density (n = 28)	Severe low bone density (n = 47)	Osteoporosis (n = 10)	Total (n = 159)	p	Padj
Geometry (mm)							
*Total area*	364 ± 84	348 ± 82	375 ± 72	316 ± 77	361 ± 81	0.15	0.30
*Trabecular area*	289 ± 82	281 ± 79	311 ± 71	255 ± 75	292 ± 79	0.14	0.90
*Cortical area*	67 ± 16	58 ± 13	53 ± 14	51 ± 19	60 ± 16	< 0.0001	< 0.0001
Volumetric densities (mg/cm^3^)							
*Total*	332 ± 65	293 ± 49	269 ± 51	285 ± 92	303 ± 66	< 0.01	< 0.0001
*Trabecular*	178 ± 40	148 ± 31	145 ± 37	139 ± 50	160 ± 41	< 0.0001	< 0.0001
*Cortical*	887 (757–971)	871 ± 59	839 ± 77	855 ± 77	876 (830–918)	0.0068	0.0008*
*Meta trabecular*	240 ± 38	207 ± 32	204 ± 36	199 ± 50	221 ± 41	< 0.0001	< 0.0001
Microarchitecture							
*Trabecular number (1/mm)*	2.06 ± 0.26	1.95 ± 0.32	1.83 ± 0.36	1.79 ± 0.46	1.96 ± 0.33	0.0001	0.0002
*Trabecular thickness (mm)*	0.072 ± 0.013	0.064 ± 0.011	0.066 ± 0.011	0.064 ± 0.014	0.068 ± 0.013	0.0035	0.0012
*Trabecular separation (mm)*	0.423 ± 0.080	0.475 ± 0.174	0.478 (0.363–0.699)	0.547 ± 0.250	0.437 (0.320–0.884)	0.0002	< 0.0001*
*Cortical thickness (mm)*	0.823 ± 0.213	0.718 ± 0.172	0.640 ± 0.182	0.659 ± 0.239	0.740 ± 0.214	< 0.0001	< 0.0001
*Cortical perimeter (mm)*	82.8 ± 10.9	81.4 ± 10.7	84.4 ± 8.9	77.9 ± 9.7	82.7 ± 10.3	0.27	0.56
*Bone volume ratio (BV/TV; %)*	14.8 ± 3.3	12.3 ± 2.6	12.1 ± 3.0	11.6 ± 4.1	13.4 ± 3.4	< 0.0001	< 0.0001

^*^Adjusted in regression analysis of the log-transformed variable

**Table 6:** Results of HRpQCT scan at tibia site, presented as mean ± SD if normally distributed or median (IQR) if non-normally distributed. P values are derived from analysis of variance (ANOVA) if normally distributed and Kruskal–Wallis test if non-normally distributed. Padj adjusted by age, gender, and BMI

	Tibia						
	Normal (n = 74)	Mild low bone density (n = 28)	Severe low bone density (n = 47)	Osteoporosis (n = 10)	Total (n = 159)	p	Padj
Geometry (mm)							
*Total area*	836 ± 154	816 ± 157	859 ± 137	749 ± 169	834 ± 151	0.18	0.26
*Trabecular area*	689 ± 153	688 ± 155	733 ± 135	641 ± 171	699 ± 150	0.22	0.84
*Cortical area*	140 ± 27	119 ± 19	115 ± 24	97 ± 20	126 ± 28	< 0.0001	< 0.0001
Volumetric densities (mg/cm^3^)							
*Total*	311 ± 56	271 ± 42	253 ± 41	248 ± 42	283 ± 56	< 0.0001	< 0.0001
*Trabecular*	182 ± 41	156 ± 32	149 ± 33	139 ± 33	165 ± 40	< 0.0001	< 0.0001
*Cortical*	889 ± 44	863 ± 39	843 ± 52	833 ± 79	867 ± 53	< 0.0001	< 0.0001
*Meta trabecular*	250 ± 41	221 ± 31	220 ± 36	207 ± 36	233 ± 40	< 0.0001	< 0.0001
Microarchitecture							
*Trabecular number (1/mm)*	1.98 ± 0.29	1.83 ± 0.32	1.72 ± 0.26	1.66 ± 0.35	1.86 ± 0.32	< 0.0001	< 0.0001
*Trabecular thickness (mm)*	0.077 ± 0.013	0.072 ± 0.014	0.073 ± 0.013	0.071 ± 0.015	0.074 ± 0.014	0.21	0.08
*Trabecular separation (mm)*	0.440 ± 0.083	0.491 ± 0.100	0.508 (0.392–0.688)	0.560 ± 0.137	0.457 (0.415–0.543)	< 0.0001	< 0.0001*
*Cortical thickness (mm)*	1.228 ± 0.259	1.056 ± 0.196	0.983 ± 0.227	0.904 ± 0.229	1.105 ± 0.264	< 0.0001	< 0.0001
*Cortical perimeter (mm)*	114.7 ± 11.2	113.4 ± 11.2	119.1 ± 23.4	108.8 ± 13.2	115.4 ± 16.0	0.19	0.73
*Bone volume ratio (BV/TV; %)*	15.2 ± 3.4	13.0 ± 2.6	12.4 ± 2.8	11.6 ± 2.7	13.8 ± 3.3	< 0.0001	< 0.0001

*Adjusted in regression analysis of the log-transformed variable

Testing for difference in HRpQCT parameters between the aBMD groups showed significant differences between the aBMD groups at the radius site in cortical area (p < 0.0001), all volumetric density measurements (p < 0.01 for total vBMD, p < 0.0001 for trabecular vBMD and p = 0.0068 for cortical vBMD, respectively), trabecular number (p = 0.0001), trabecular thickness (p = 0.0035), trabecular separation (p = 0.0002), cortical thickness (p < 0.0001), and bone volume ratio (p < 0.0001). After adjustment for age, sex, and BMI, the associations remained except for trabecular thickness which was borderline significantly reduced (p = 0.0035). In post hoc analyses, the volumetric densities, trabecular number, trabecular thickness, cortical thickness, and bone volume ratio showed to be lower in the groups with lower aBMD (e.g., low bone mineral density and osteoporosis), whereas trabecular separation was higher in the groups with lower aBMD.

Similar significant differences between aBMD groups that were present at the tibia site were cortical area (p < 0.0001), all volumetric density measurements (p < 0.0001 for all), trabecular number (p < 0.0001), trabecular separation (p < 0.0001), cortical thickness (p < 0.0001), and bone volume ratio (p < 0.0001).

After adjustment for age, sex, and BMI, the associations remained. In post hoc analyses, the volumetric densities, trabecular number, cortical thickness, and bone volume ratio showed to be lower in the groups with lower aBMD (e.g., low bone mineral density and osteoporosis), whereas trabecular separation was higher in the groups with lower aBMD.

## Discussion

We evaluated aBMD as well as peripheral vBMD and bone microarchitecture in PLHIV. The prevalence of low bone density by DXA was 47% (29% with T-score ≤ − 1.5).

This study is among the first studies reporting HRpQCT data in PLHIV and, to our knowledge, the largest cohort of PLHIV yet investigated by this method. Lower aBMD was associated with lower cortical area at both the tibia and radius, but not with significant differences in total or trabecular area. Lower aBMD was also associated with lower volumetric densities at both sites and generally deteriorated microarchitecture parameters at both sites. Microarchitecture parameters were affected at both trabecular and cortical compartments, but mainly trabecular compartments.

A normal mean CD4 cell count of 690 (SD ± 332) with no significant difference between aBMD groups suggests that our study population is a well-treated HIV population and no contribution of CD4 cell number to the differences in BMD seen.However, it should be noted that BMD reflects accumulated effect on bone mass over years, whereas CD4 cell number reflects the current situation. Bone turnover marker analysis showed a trend toward a higher CTX level in PLHIV with lower BMD (p = 0.05) but no significant difference in P1NP levels between BMD groups.

Our results indicate that low bone density and osteoporosis in PLHIV could partly be explained by low BMI. A meta-analysis has found low BMI (among others) to be independently associated with lower BMD in postmenopausal women living with HIV [27]. Data from the COCOMO cohort show a higher prevalence of very low BMD in PLVHIV compared to controls using quantitative CT of the thoracic spine; however, the difference between the groups attenuated when the analysis was adjusted for traditional osteoporosis risk factors, underlining that non-HIV-related factors are important contributors to bone health in PLHIV [28].

The design of our study did not allow assessment of whether HIV per se, ART in general or a specific ART regimen, contribute to the increased fracture risk observed in PLHIV. However, a combination of HIV infection itself and ART could potentially influence fracture risk since 1) ART-naïve PLHIV have lower BMD compared to uninfected controls [29], 2) PLHIV treated with ART have a higher prevalence of reduced BMD compared to ART naïve [30], and 3) there is an association of TDF treatment with increased fracture risk and BMD reduction in both PLHIV and uninfected controls [31]. Hansen et al. [25] found significantly increased cumulative incidence of fragility fractures after ART initiation. A direct comparison of the effect of HIV itself in treatment naïve compared to controls and ART treated is difficult since it is recommended to treat all PLHIV regardless of CD4 + T-cell number.

Strengths of this study include the large number of participants completing the HRpQCT scan. There are also limitations to this study including no specific uninfected control group and the observational design that only allows identification of associations but not causality. Also, 92% of our participants were Caucasian. Microarchitectural advantage has been documented for both Asians [32] and Africans [33] compared to Caucasians and this may lead to underestimation of differences in our population.

In conclusion, we observed alterations in cortical area, vBMD, and most microarchitecture parameters between groups defined by aBMD in PLHIV. Low BMI is associated with alterations in BMD and microarchitecture. Prospective studies are needed to further establish the contribution of these to bone fragility and fracture risk in the aging HIV population.

## Supplementary Information

Below is the link to the electronic supplementary material.

Supplementary file1 (PDF 23 KB)

## Data Availability

The datasets presented in this article are not readily available due to the regulations of the Danish Data Protection Agency. Requests to access the datasets should be directed to the corresponding author.

## Abbreviations

AIDS: Acquired immune deficiency syndrome
ART: Antiretroviral treatment
BMI: Body mass index
BMD: Bone mineral density
CTX: C-terminal telopeptide of type I collagen
DXA: Dual energy x-ray absorptiometry
FN: Femoral neck
FRAX: The fracture risk assessment tool
HBV: Hepatitis B virus
HCV: Hepatitis C virus
HIV: Human immunodeficiency virus
HRpQCT: High-resolution peripheral quantitative computed tomography
LS: Lumbar spine
MSM: Men who have sex with men
P1NP: N-terminal propeptide from type 1 collagen
PTH: Parathyroid hormone
PLHIV: People living with HIV
TAF: Tenofovir alafenamide
TDF: Tenofovir disoproxil fumarate
TH: Total hip
