# Supplementary material for: Investigation of Osteoporosis in Persons Living with Human Immunodeficiency Virus: The HOST Study
Source: Calcif Tissue Int. 2025 Apr 25;116(1):64. doi: 10.1007/s00223-025-01368-8 (PMC12031905; doi:10.1007/s00223-025-01368-8)
Supplement: Supplementary file 1 — Supplementary file1 (PDF 23 KB) [file 223_2025_1368_MOESM1_ESM.docx]

## Investigation of Osteoporosis in Persons Living with Human Immunodeficiency Virus: The HOST study

Simone Bruhn Rosendahl (SBR)^1,2^, Jakob Starup-Linde (JSL)^1^, Merete Storgaard (MS)^2^, Bente Langdahl (BL)^1^

1) Department of Endocrinology and Internal Medicine, Aarhus University Hospital, Aarhus, Denmark

2) Department of Infectious Diseases, Aarhus University Hospital, Aarhus, Denmark

Corresponding author: Simone Bruhn Rosendahl. E-mail: simore@rm.dk

Journal: Calcified Tissue International

## Supplemental material

Supplemental table 1.

*Bone markers CTX and P1NP for fasting, non-fasting and total study population. Presented as mean ± SD if normally distributed or median (IQR) if non-normally distributed. P values are derived from Student’s t-test (if normally distributed) or Wilcoxon rank-sum test (if non-normally distributed) of difference in CTX and P1NP values, respectively, between fasting and non-fasting participants.*

|  | Normal | Mild osteopenia | Severe osteopenia | Osteoporosis | Total |
| --- | --- | --- | --- | --- | --- |
| *CTX fasting(ng/mL)* | 0.355 (0.238-0.489)  (n=58) | 0.417 ± 0.149  (n=19) | 0.455 ± 0.181  (n=34) | 0.584 ± 0.204  (n=8) | 0.429 ± 0.183  (n=119) |
| *CTX non-fasting(ng/mL)* | 0.288 ± 0.146  (n=17) | 0.378 ± 0.255  (n=9) | 0.372 ± 0.185  (n=13) | 0.225 ± 0.152  (n=2) | 0.331 ± 0.186  (n=41) |
| *CTX all (ng/mL)* | 0.344 (0.226-0.464)  (n=75) | 0.404 ± 0.186  (n=28) | 0.432 ± 0.184  (n=47) | 0.512 ± 0.240  (n=10) | 0.404 ± 0.189  (n=160) |
| *p (fasting vs non-fasting)* | 0.03 | 0.62 | 0.17 | 0.05 | 0.004 |
|  |  |  |  |  |  |
| *P1NP fasting (ng/mL)* | 52.33 (41.87-66.61)  (n=58) | 63.84 ± 35.18  (n=19) | 59.75 ± 24.80  (n=34) | 70.73 ± 26.78  (n=8) | 60.56 ± 28.95  (n=119) |
| *P1NP non-fasting (ng/mL)* | 52.65 ± 17.87  (n=17) | 65.90 ± 29.23  (n=9) | 58.81 ± 22.33  (n=13) | 76.44 ± 37.00  (n=2) | 58.67 ± 22.92  (n=41) |
| *P1NP all (ng/mL)* | 51.19 (39.21-66.48)  (n=75) | 64.50 ± 32.85  (n=28) | 59.49 ± 23.91  (n=47) | 71.87 ± 26.76  (n=10) | 60.08 ± 27.48  (n=160) |
| *p (fasting vs non-fasting)* | 0.68 | 0.88 | 0.91 | 0.80 | 0.71 |
